# Analysis of the acute phase responses of Serum Amyloid A, Haptoglobin and Type 1 Interferon in cattle experimentally infected with foot-and-mouth disease virus serotype O

**DOI:** 10.1186/1297-9716-42-66

**Published:** 2011-05-18

**Authors:** Carolina Stenfeldt, Peter MH Heegaard, Anders Stockmarr, Kirsten Tjørnehøj, Graham J Belsham

**Affiliations:** 1 National Veterinary Institute, Technical University of Denmark (DTU-Vet), Division of Virology, Lindholm, DK-4771 Kalvehave, Denmark; 2 National Veterinary Institute, Technical University of Denmark (DTU-Vet), Division of Veterinary diagnostics and research, Bülowsvej 27, DK-1790 Copenhagen, Denmark

## Abstract

A series of challenge experiments were performed in order to investigate the acute phase responses to foot-and-mouth disease virus (FMDV) infection in cattle and possible implications for the development of persistently infected "carriers". The host response to infection was investigated through measurements of the concentrations of the acute phase proteins (APPs) serum amyloid A (SAA) and haptoglobin (HP), as well as the bioactivity of type 1 interferon (IFN) in serum of infected animals. Results were based on measurements from a total of 36 infected animals of which 24 were kept for observational periods exceeding 28 days in order to determine the carrier-status of individual animals. The systemic host response to FMDV in infected animals was evaluated in comparison to similar measurements in sera from 6 mock-inoculated control animals.

There was a significant increase in serum concentrations of both APPs and type 1 IFN in infected animals coinciding with the onset of viremia and clinical disease. The measured parameters declined to baseline levels within 21 days after inoculation, indicating that there was no systemically measurable inflammatory reaction related to the carrier state of FMD. There was a statistically significant difference in the HP response between carriers and non-carriers with a lower response in the animals that subsequently developed into FMDV carriers. It was concluded that the induction of SAA, HP and type 1 IFN in serum can be used as markers of acute infection by FMDV in cattle.

## Introduction

Foot-and-Mouth-Disease (FMD) is a highly contagious viral disease which affects cloven-hoofed animals including cattle, sheep and pigs, with substantial financial implications for affected countries. Severity of clinical disease varies between common domestic species, with pigs developing severe clinical illness, followed by cattle showing obvious but less severe clinical signs, whilst the clinical course of the infection in sheep may be very mild [1].

Foot-and-mouth disease virus (FMDV) is a positive stranded RNA virus. It is the prototype virus of the *Aphthovirus *genus within the picornavirus family. The viral genome includes a single large open reading frame encoding a polyprotein which is cleaved by virus-encoded proteases giving rise to structural and non-structural proteins needed for replication and assembly of new virus particles [2].

The predominant site of initial FMDV replication within infected animals is thought to be located within the epithelia of the pharyngeal mucosa [3-6], or alternatively within the lungs [7,8]. From here the virus spreads via the lymphatics and vascular system to the peripheral sites of secondary replication, characterized by the presence of stratified cornified squamous epithelia, such as the coronary bands and oral cavity [3]. Infected cattle develop transient viremia lasting for 2-3 days, which is effectively counteracted by the development of circulating anti-FMDV antibodies. The clinical disease follows a rapid time course and is typically manifested by a sudden rise in body temperature and development of vesicular lesions at peripheral areas of viral replication. Affected animals may display varying degrees of salivation, inappetence and lameness corresponding to the severity of lesions. The clinical course of the infection usually subsides within 7-14 days but the potential development of persistently infected carrier-animals creates further complications for disease control. FMDV carriers are defined as animals with asymptomatic, intermittent, presence of infectious virus in oropharyngeal fluid more than 28 days post infection (dpi) [9].

It is believed that in these animals (approximately 50% of infected cattle), FMDV is capable of persisting, at a low level, within pharyngeal epithelial cells [10,11], or as intact, but largely quiescent, viral particles within germinal centers in pharyngeal tissues [12]. Since carrier animals are a potential source of infectious virus, their presence is unacceptable in areas free of FMD.

Development of the carrier state is unaffected by the presence of neutralizing antibodies in the circulation. Thus, both animals that are immunologically naïve at the time of exposure to FMDV, as well as those with circulating antibodies due to vaccination or previous exposure to the virus can become FMDV-carriers, regardless of pre-occurring clinical disease [9,10,13]. It is known that FMDV carriers do exhibit a measurable adaptive immune-response comparable to that of animals that clear the infection [14] but there is still a significant lack of knowledge regarding the innate immune-response to FMD in cattle. The duration of the carrier state varies between species, with the longest duration recorded in African buffaloes (5 years), followed by cattle (2 years) and sheep (9 months) [15-18]. Pigs do not become carriers [14].

The innate immune response induced by a viral infection in the upper respiratory tract is characterized by initial activation of peripheral primary effector cells which function to initiate a local inflammatory response, priming and recruiting activators of the cellular immune response. Macrophages present in the respiratory tract produce pro-inflammatory cytokines such as tumor necrosis factor-α, interleukin-1 and interferon (IFN) upon stimulation of pattern recognizing surface receptors, causing alterations in local vascular walls, and providing recruitment and activating stimuli to antigen presenting cells and phagocytes [19,20].

Type 1 IFNs are also known as viral IFNs and include interferon-α and -β. These IFNs are secreted by virus infected cells with the function of blocking spread of virus to uninfected cells by inducing alterations in gene transcription and protein synthesis following their interaction with common cell-surface interferon receptors [20,21]. It has been proposed that type 1 IFNs may have an important role in the host response to FMDV [14,22,23] and that the ability of the virus to induce an IFN response may be related to the pathogenicity of different isolates of FMDV [24,25].

Locally produced pro-inflammatory mediators also cause alterations in hepatic metabolism, inducing the production of various acute phase proteins (APPs) in the circulation [19]. APPs have been defined as proteins for which serum concentrations are significantly altered in acutely infected animals, compared to that of animals which are clinically healthy [26]. The proteomic pattern of the acute phase response varies between animal species and also depends upon the pathogen responsible for inducing the response [27,28].

Previous studies have shown a correlation between increased serum levels of bovine haptoglobin (HP) and the onset of clinical disease in bovine respiratory disease [26], as well as in infections with bovine respiratory syncytial virus [29]. Increased serum concentrations of HP in cattle acutely infected with FMDV through aerosol exposure, have also been demonstrated [30].

The production of HP and serum amyloid A (SAA) are considered reliable indicators of acute inflammation caused by various infectious agents in cattle [31].

The aim of this study was to investigate the innate immune response to infection with FMDV in cattle by measuring systemic levels of acute phase proteins SAA and HP as well as the biological activity of type 1 IFN in serum. Results from these measurements were evaluated in relation to the timing of the appearance of clinical signs of disease as well as the detection of viremia and circulating antibodies. Measured parameters were further analyzed statistically in order to evaluate whether there was any detectable difference in host response between the animals that developed into persistently infected carrier animals and those that were efficient in clearing the infection completely.

## Materials and methods

### Animal experiments and samples

Animal experiments were performed in high containment research facilities at DTU-Vet, Lindholm Island, Denmark, in accordance with the requirements of the Danish Animal Experiments Inspectorate (License 2003/561-742; 2008/561-1541).

Three independent experiments were included in this study. The animals used were 4-5 month old steers of mixed-Holstein breed. The first experiment (FMD 2008) was performed with 12 animals (six inoculated and six in direct contact). In each of the two subsequent experiments (FMD 2010 a + b), three uninfected control animals, kept in a separate isolation unit, were used in addition to the twelve test animals. Results from this study are thus based on measurements in 36 FMDV infected animals and 6 uninfected controls. Apart from observations of clinical signs of disease and measurements of standard para-clinical parameters such as virus excretion and development of circulating antibodies (see description of protocol below), all three experiments included assays for the serum concentrations of the APPs (SAA and HP) as well as for the bioactivity of type 1 IFN in serum. In the first experiment (FMD 2008) animals were kept under observation for a total of 100 dpi, whilst the experimental periods for the two subsequent experiments (FMD 2010 a + b) were 35 and 14 dpi respectively. The following protocol was used in all three experiments.

All animals were pre-treated using a broad spectrum antibiotic (enrofloxacin; "Baytril^©^"; 2.5 mg/kg) for four days upon arrival in order to reduce the level of any existing bacterial infection. Animals were subsequently allowed a period of acclimatization for a minimum of 10 days, and were further treated with β-lactam penicillin ("streptocillin^©^"; 5 mL/100 kg) for four days around inoculation to avoid interference caused by any bacterial infections.

Six animals were inoculated with FMDV O UKG 34/2001 (original inoculum obtained from the Institute for Animal Health (IAH)-Pirbright, and then passaged once in cattle) using subepidermo-lingual injection, each animal receiving approximately 10^6.9 ^TCID_50 _in a volume of 0.5 mL administered at 6 to 8 injection sites at the base of the tongue. A standard protocol for sedation consisting of intravenous injection of Xylazinhydrochloride (Rompun^© ^2%; 1.5 mL/animal) was used for inoculation and the sedation was reversed through intravenous administration of Antipamezol (Antisedan^©^; 0.5 mL/animal). Six other animals were kept in continuous direct contact with the inoculated animals, with two inoculated plus two contact animals in each pen and with the stable facilities allowing direct contact between animals in separate pens.

All animals were monitored daily, with measurements of rectal temperature and observation of clinical signs. Serum samples were collected daily from 3 days prior to inoculation and throughout the first two weeks of the experiments and thereafter on a weekly basis throughout the remaining part of the experiment. Blood samples were stored at 4°C overnight, before centrifugation and the sera were subsequently stored at -70°C.

Samples of oropharyngeal fluid (probang samples) [32] for quantification of virus excretion, in order to define the carrier status of individual animals [33], were collected prior to inoculation, once daily during the first week after inoculation, then every other day throughout the second week and on a weekly basis subsequently. Probang samples were collected at closer intervals from post infection day (pid) 28 until termination of the experiment so that the carrier status of individual animals was based on the analysis of a minimum of four samples. Control animals were handled and sampled following a protocol similar to that used for infected animals. The animals were sedated and "mock-inoculated" using phosphate buffer, in order to mimic the procedure used for inoculation and sample collection in the test groups.

### Quantification of Acute Phase Proteins in serum

#### Serum Amyloid A

Serum concentrations of SAA were determined using a sandwich-ELISA kit (Tridelta Developments Ltd. Maynooth, County Kildare, Ireland). Samples were analyzed at a dilution of 1:500, according to the manufacturer's instructions. A standard dilution of the SAA calibrator included in the kit was added in duplicate to each of the ELISA plates and SAA concentrations in the sera were determined from the standard curves.

#### Haptoglobin

Serum concentrations of HP were determined using a sandwich-ELISA, following a protocol described in detail previously [29] using monoclonal anti-bovine haptoglobin antibodies kindly provided by Dr Philip Griebel, University of Saskatchewan, Canada.

Briefly, microtitre plates were coated with anti-bovine haptoglobin ascites fluid in carbonate buffer. Serum samples were tested at three dilutions (1:100; 1:300; 1:900), together with a calibration standard of acute phase serum with known concentrations of HP. Bound antigen was detected using biotinylated anti-bovine haptoglobin Mabs, by staining with TMB reagent in citrate buffer. The colour development was terminated by addition of sulfuric acid, and OD-values were read using a standard ELISA reader at wavelengths of 450/650 nm.

### Quantification of the bioactivity of Type 1 IFN in serum

Biological activity of type 1 IFN was quantified with an Mx/CAT reporter gene assay [34], using the transfected MDBK-t2 cell line kindly provided by Dr Bryan Charleston (IAH-Compton, UK). Briefly, the cells were seeded in 24-well plates and cultured in 1500 μL of medium (EMEM with blasticidin (10 μg/mL), penicillin (100 IU/mL), streptomycin (100 μg/mL) and 10% FCS).

Serum samples, diluted 1:5 in cell culture medium containing 2% FCS, were added to the cells and incubated overnight at 37°C. All samples were tested in duplicate, in parallel with standard concentrations (0.125 to 90 IU/mL) of recombinant human IFN-α (Invitrogen). Cells were harvested and CAT-expression was quantified using a commercial CAT-ELISA kit (Roche Applied Science) following the manufacturer's instructions.

### Quantification of anti-FMDV antibodies using a solid phase blocking ELISA

Serum concentrations of FMD specific antibodies were measured using a serotype-specific solid phase blocking ELISA [35]. In brief, microtitre plates were coated with guinea-pig immune sera raised against FMDV O Manissa before addition of inactivated FMDV antigen. Samples of the bovine sera were then added, incubated overnight and then rabbit anti-FMDV serotype O serum was added and the bound antibodies were detected using horseradish-peroxidase conjugated porcine anti-rabbit IgG. All serum samples were initially screened at a dilution of 1:5 and positive samples (blocking percentage >50%) were analyzed in a two-fold titration starting at a dilution of 1:10 to allow determination of antibody titers.

### Quantification of viremia and excretion of FMDV RNA in probang samples

Quantification of FMDV RNA in serum and probang samples was performed using quantitative RT-PCR [33]. Total RNA was extracted using a MagNa Pure LC Total Nucleic Acid Isolation Kit (Roche) with an automated robotic workstation (Roche) from 200 μL of sample, according to the manufacturer's instructions. Each RNA sample was eluted in a volume of 50 μL and stored at -70°C until further processing. Reverse transcription of FMDV RNA was carried out using 6 μL of extracted RNA in a total volume of 15 μL, using a TaqMan RT kit with random hexamer primers (Applied Biosystems) at 48°C for 45 min and 95°C for 5 min. Then 7 μL of each cDNA was mixed with 18 μL of 2x TaqMan universal PCR mastermix (Applied Biosystems) containing 22.5 pmol of each primer and 5 pmol of fluorescently labeled probe. PCR amplification was carried out for 50 cycles, in an Applied Biosystems Model Mx 3005P Thermal cycler and analysed using MxPro qPCR software.

### Statistics

APP responses were log-transformed in order to reduce fluctuations in data and to ensure a normal distribution of residuals. A response measure representing the area under the curve was constructed, where linear interpolation between points of observations was used, and a slight extension at end points to put equal weight on observations evenly spaced in time. APP responses were analyzed with a standard random effects model [36], where the identification of the three sequentially performed experiments ("survey") was entered as a random effect. Fixed effects were Carrier status ("Carrier"), route of infection ("status": inoculated, contact or control), level of viremia and maximum anti-FMDV antibody titre. The effect of the carrier-status of individual animals was modeled as a covariate with the values 1/0 representing carrier/non-carrier. Tests were performed as likelihood ratio tests, according to the following model,

where β represents coefficients of effect and η represents the standard deviation between experiments (i).

Model control was performed with standard techniques [36]. For the APP SAA, the model control revealed that criteria were not met for the response measure, and the tests were subsequently performed as z-tests (continuous fixed effects). All analyses were performed with Splus^© ^software version 6.1 (Insightful Corp. 2002).

## Results

### Clinical symptoms

**Figure 1:**
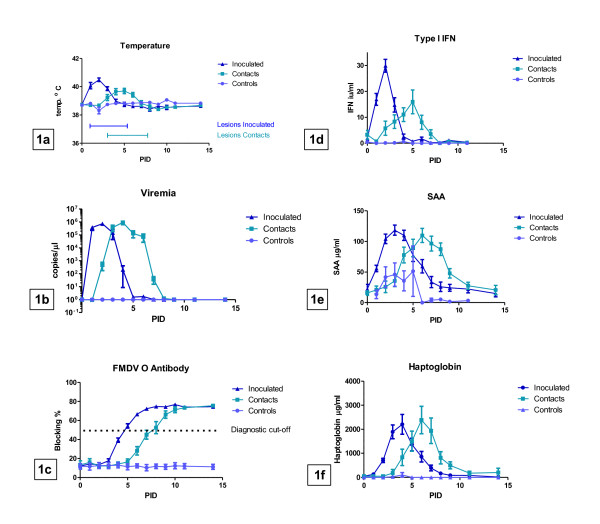
**Characterization of clinical signs and acute phase responses in FMDV-infected cattle**. The results shown are the mean values (+/- S.E.M) for the indicated parameters from three separate experiments each including 12 or 15 cattle (6 inoculated with FMDV, 6 in-contact and 3 uninfected controls in 2 of the experiments). a) Temperature and timing of occurrence of characteristic vesicular lesions within the oral cavity. b) Viremia measured by qRT-PCR expressed as copies of FMDV genome/μL of serum c) anti-FMDV (type O) antibodies in serum expressed as blocking percentage derived from a solid phase blocking ELISA d) Bioactivity of type 1 IFN in serum (iu/mL) E) Haptoglobin in serum (μg/mL) f) Serum Amyloid A in serum (μg/mL).

All animals included in the test groups developed mild to moderate clinical signs of FMD, with an increase in body temperature (Figure 1a) and vesicular lesions in the oral cavity being the most prominent findings occurring in all individuals. Less than half of the animals developed vesicular lesions on the feet and only one individual (out of 36) developed clearly visible lameness. Inoculated animals developed clinical signs of FMD at pid 1-2, whilst onset of clinical disease in contact animals was observed from pid 2-5. The animals showed no loss in appetite and all animals recovered from clinical disease within approximately seven days, without further complications, and without the need of supportive medical treatment.

### Viremia and development of antibodies

FMDV RNA was detectable in serum from all infected animals for a period of four to seven days (Figure 1b). Inoculated animals showed significant viremia at pid 1, reflecting the very rapid replication and systemic spread of FMDV. Anti-FMDV serotype O antibodies in serum reached the diagnostic cut-off level, defined as a blocking percentage of 50%, at around pid 4 to 5 in inoculated animals and at pid 7 to 9 in the contact group (Figure 1c). Appearance of circulating antibodies was accompanied by a rapid reduction in viremia. There were no detectable differences in the level of viremia (presented as the number of copies of FMDV genome per μL of serum) or the anti-FMDV O antibody titres (data not shown) between directly inoculated and contact infected animals, or between carriers and non-carriers.

### Prevalence of Carriers

Carrier status was determined on the basis of detection of FMDV RNA, using qRT-PCR, in probang samples beyond 28 dpi [33]. An animal was regarded as being a carrier following detection of at least two positive probang samples (Ct value <40) out of a minimum of four samples collected beyond pid 28. Detailed information on the extent of virus excretion in probang samples in carriers and non-carriers has been included in a separate report (Stenfeldt and Belsham, submitted). In summary, animals identified as FMDV carriers had detectable levels of FMDV RNA (Ct <40) in probang samples harvested throughout the entire length of the experimental period whereas FMDV RNA became undetectable in probang samples from the non-carriers by approximately 14 dpi. One of the experiments (FMD 2010b) was terminated at 14 dpi, for technical reasons, and so it was therefore not possible to determine carrier-status in this experiment. In the remaining two experiments (FMD 2008 and FMD 2010a) the prevalence of carriers was 6/12 (50%) and 3/12 (25%) respectively (see Table 1), consistent with what has been found previously in experimental infections with FMDV [1,17,37].

**Table 1:** Distribution of FMDV carriers within the directly inoculated and contact infected animals.

Experiment ID	Number of animals (Inoculated: Contact)	Carriers (Inoculated: Contact)	Non-Carriers (Inoculated: Contact)
**FMD 2008**	12 (6:6)	6 (4:2)	6 (2:4)
**FMD 2010 a**	12 (6:6)	3 (1:2)	9 (5:4)

### Acute phase proteins

The serum levels of APPs showed a consistently occurring peak, coinciding with the onset of clinical symptoms (Figure 1e-f). Directly inoculated and contact animals showed similar responses when the areas under the curve (AUC) were compared for the serum concentrations of SAA during the observation period of 14 days after infection. For the HP-response, there was however, a small but statistically significant difference (*p *= 0.04) between inoculated and contact animals, with a larger response in the directly inoculated animals. There was a very marked and statistically significant (*p *< 0.0001) difference in the AUC values for both SAA and HP when comparing test-groups (FMDV inoculated and contacts) to the uninfected control animals.

**Figure 2:**
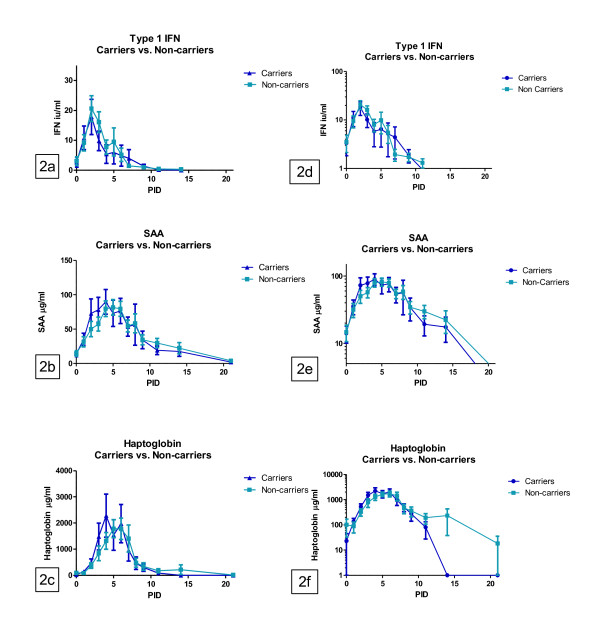
**Comparison of acute phase protein and IFN measurements in carrier and non-carrier cattle on linear (a-c) and logarithmic scale (d-f)**. a+d) Bioactivity of type 1 IFN in serum (iu/mL). b+e) Serum amyloid A in serum (μg/mL). c+f) Haptoglobin in serum (μg/mL).

It is interesting to note that, there was also a statistically significant difference (*P *= 0.015) in the AUC values for the serum concentration of HP between the animals that became carriers and the non-carriers, with lower values in the carrier animals (Figure 2f; Tables 2 and 3). The difference in the HP-response between carriers and non-carriers was unrelated to, and could therefore not be explained by, the observed difference between inoculated and contact-infected animals for this parameter. There was no difference in the AUC values for serum concentration of SAA between carriers and non-carriers (Table 2).

**Table 2:** Summary of P-values for comparisons of the AUC values of serum concentrations of APPs and IFN during a test period of 14 dpi.

	Inoculated and Contacts vs. Controls	Inoculated vs. Contacts	Carriers vs. Non-Carriers
**SAA**	<0.0001***	0.61	0.49
**IFN**	<0.0001***	0.43	0.27
**Haptoglobin**	<0.0001***	0.04*	0.015*

Levels of significance: ***= *p *< 0.0001; **= *p *< 0.001 *= *p *< 0.05.

**Table 3:** Coefficients of effect (β) for contribution to AUC values of serum HP of individual animals for statistically significant effects: carrier-status ("Carrier": carrier (1)/non-carrier (0)), route of infection ("Status": inoculated/contact-infected (uninfected controls are not included in this analysis as these do not become carriers))

Final model	**log(Hp**_**ij**_**) = β**_**C **_**Carrier**_**ij **_**+ β**_**Statusij **_**+ηY**_**Surveyi **_**+ ε**_**ij**_**, i = 1:3,j = 1:n**_**i**_,		
		**β**_**C**_	**95% confidence interval**
**Carrier Status**	Carrier	-1.14	-1.98 to -0.3
	Non-carrier	0	(NA)

		**β**_**Status**_	**95% confidence interval**
**Route of infection**	Inoculated	4.52	3.19 to 5.85
	Contact	3.65	2.34 to 4.96

η represents standard deviation between experiments (i).

### Type 1 IFN

Type 1 IFN bioactivity, as measured by the activation of the Mx promoter in the MDBK-t2 cells, showed a reaction pattern similar to the APPs, with a marked peak at the onset of clinical symptoms and thus significant differences in response between the infected test-groups and uninfected control animals. Contact animals seemed to show slightly lower peaks in IFN response than directly inoculated animals (Figure 1d), although this difference was not statistically significant (Table 2). There was no difference in the AUC values for the type 1 IFN bioactivity between carriers and non-carriers (Table 2).

## Discussion

The observed progression of the clinical infection, including the level of viremia and titres of circulating antibodies, was similar to what has been described previously for infection with FMDV O UKG/34 2001 in cattle [38,39].

Measurements of serum concentrations of the APPs, SAA and HP, indicated a rapid acute phase response which coincided with the appearance of clinical signs of disease. It has previously been proposed that the sensitivity of SAA as a marker of infection could be higher than that of HP [31]. In our studies we observed a relatively high basal level and measurable changes in the level of SAA in some of the mock-inoculated control animals, although by no means approaching the significantly up-regulated concentrations observed in animals infected with FMDV. In contrast to this, we recorded very low basal levels in the HP measurements within uninfected animals, whilst both APP markers showed a clear response with significantly increased concentrations within the sera from infected animals. It has been shown that changes in SAA levels are more easily induced by physical stress than for HP [40]. It may be that the procedures used for handling of animals for sampling, including the protocol for sedation used for inoculation, could be responsible for inducing a significant stress response in the animals, which might be the cause of the observed SAA response in some of the mock-inoculated control animals.

The acute phase response to FMD in cattle has previously been investigated by Hofner et al. [30] using measurements of HP in sera from cattle that were infected with FMDV O BFS 1860 following aerosol exposure. It was reported that an increase in serum concentrations of HP occurred 8-9 days after virus exposure and 1-3 days after the onset of viremia and appearance of clinical signs. It was concluded that there was no measurable rise in HP during the "pre-viremic" phase of FMD infection. Furthermore, it was proposed that this finding was indicative of little or no tissue damage during initial replication of FMDV in the pharyngeal epithelia.

The clinical course of FMD in our experiments differed slightly from what was described by Hofner et al. [30]. We observed a more rapid course of infection, with simultaneous appearance of viremia and clinical signs of disease, including an increase in body temperature, in both directly inoculated and in contact animals. We also detected an increase in serum concentrations of HP as well as SAA, which for the majority of the animals began at the same time as the observed onset of clinical disease and appearance of viremia. Thus, there was no apparent lag phase between the rise in body temperature and the measurable acute phase response in serum.

The differences in clinical observations may partly be a result of the different means of inoculation used in the two studies; direct inoculation in our studies versus aerosol exposure in the study performed by Hofner et al. [30]. However, in our studies, we also saw similar timing of reactions in animals infected by contact exposure as in those that had been directly inoculated with simultaneous detection of viremia and clinical parameters. Thus, the observed variations in results are more likely to be derived from differences in sensitivity of the different assays used for quantification of HP and detection of viremia in the two studies. In the earlier study, the HP concentration was quantified through an assay measuring hemoglobin binding capacity while viremia was detected by inoculation of primary cell cultures, whereas we measured HP with a specific and sensitive ELISA assay and viremia with a quantitative RT-PCR.

The measured changes in the bioactivity of type 1 IFN in serum followed a similar pattern to what was recorded for the APPs, with measurable peaks in activity of IFN in the sera at the onset of clinical disease. There seemed to be a slight, although not statistically significant, difference in maximum peak levels of IFN bioactivity in inoculated versus contact animals. Animals in the contact groups acquired the infection through continuous exposure to virus excreted by infected animals, whereas the inoculated animals received a single high level dose of virus in the tongue. The different level and route of virus access to the primary site of replication is likely to cause a variation in the timing and synchronization of the initial cellular immune response responsible for the measured cytokine response.

It is believed that the acute phase response of the liver is induced in response to the presence of inflammatory cytokines in the circulation [19]. Our results indicate near simultaneous timing for the onset of induction of APPs and type 1 IFN in both inoculated and contact-infected animals, with the rate of the initial response in SAA slightly exceeding that of HP. It is possible that the very rapid onset of viremia, with high levels of virus in the blood seen as early as pid 1 in the inoculated animals, could in some way influence the hepatic APP response in a more direct manner.

Type 1 IFN is produced by virus-infected cells, as well as by inflammatory cells (macrophages and cells of the dendritic cell lineage) present within the circulation and peripheral tissues [41,42]. Previous studies have shown an up-regulation of IFN-α and-β mRNAs within epithelial cells harvested from FMDV lesions of the oral cavity [42]. We have attempted to quantify IFN bioactivity in samples of oropharyngeal scrapings (probang samples) without any success (data not shown), possibly indicating that the activity of IFN is somehow inhibited in this type of sample.

Recent studies in cell culture have demonstrated that the leader protease (Lpro) of FMDV is capable of down-regulation of transcription of IFN-β through interaction with NFκB [25,43]. The Lpro also inhibits host cell protein synthesis [44,45], thus within FMDV-infected cells the expression of IFN mRNA and its translation should be blocked. This may suggest that the IFN present in serum is not produced by cells that are actually infected with FMDV but by populations of immune cells that react upon the presence of virus through activation of pattern recognizing receptors. Plasmacytoid dendritic cells, also referred to as natural interferon producing cells (NIPC), are known to produce high levels of type I IFN in response to virus infection [46]. It is, however, suggested that it is necessary for non-enveloped viruses such as FMDV to be in a complex bound by antibody in order to induce IFN production by cells of this lineage [47]. This putative relationship does not match the timing of events observed in our study as the increase in type 1 IFN activity in serum was detected very early (pid 1-2) which significantly precedes the rise in circulating anti FMDV-antibody. Investigations of the cellular source of the systemically measurable rise in IFN activity might help to further elucidate the interactions between the virus and the immune response of the host. Previous studies have reported an increase in IFN-α mRNA levels in nasal associated lymphoid tissue harvested from the pharyngeal region of calves acutely infected with FMDV serotype O [48]. The same study also reported a difference in the expression of tumor necrosis factor-α (TNF-α) between carriers and non-carriers in similar samples collected from a total of six animals at 62 dpi. We have been unable to find evidence of an up-regulation of IFN mRNA levels in samples of pharyngeal mucosa collected at sequential time points during infection. However, we did corroborate the previous report of a difference in TNF-α mRNA expression in the pharyngeal epithelium of FMDV-carriers and non-carriers during the late phase of infection (> pid 28) (Stenfeldt et al., submitted).

Alsemgeest et al. [49] compared serum concentrations of SAA and HP in acute and chronic inflammation in cattle. It was found that the HP/SAA ratio differed during different stages of infection, with the serum concentration of SAA exceeding that of HP in acute inflammation and the opposite occurring during chronic inflammation. In our studies, we saw that concentrations of both SAA and HP declined to baseline-levels by pid 14-21, regardless of whether the animal was persistently infected with FMDV or not. These findings indicate that the carrier status in FMDV infection in cattle is not accompanied by a state of systemically measurable chronic inflammation.

There was no measurable difference in the serum reactions of SAA and type 1 IFN between infected animals which became carriers or non-carriers. For HP, however, there was a statistical difference in the AUC values between animals which were identified subsequently as carriers and non-carriers with a lower HP level in carriers. The observed difference in the HP response is most clearly visible at the latter part of the timescale when viewing the log-transformed data (Figure 2f). This data may be an indication of slightly prolonged reaction in HP in the sera of the animals that were successful in clearing the infection completely. When extending the observational period to include analysis of sera collected at pid 21 it was, however, clear that the serum concentration of HP did decline to baseline levels (below the assay detection limit) in all animals regardless of carrier-status.

The observed difference in HP-response between directly inoculated and contact infected animals, which only just reached statistical significance (*p *= 0.04), could possibly (as previously discussed for the IFN-response), be explained by the differences in the routes and timing of virus exposure.

The relationship between the HP response and the development of FMDV carrier animals should be further investigated in future experiments to elucidate the nature of the apparent difference in the acute host response to FMDV between carriers and non-carriers.

There might also seem to be a slight difference in the time course of SAA and HP responses between animals which became carriers and non-carriers, with a more rapid pattern of reactions in the carrier-group. This apparent difference is, however, most likely caused by a variation in the timing of the reactions between inoculated and contact animals, with a relatively larger number of inoculated animals, compared to contact-infected animals within the group of carriers. However, the route of infection (directly inoculated versus contact-infected) did not have any effect on whether the animal developed into a persistently infected carrier or not (Table 1).

Observations from our experiments indicate that combined measurements of serum concentrations of SAA and HP can be used as markers of an acute systemic response to FMDV infection in cattle. This could, for example, be of value during vaccine studies or transmission experiments with FMDV isolates of low virulence, where it would be of interest to detect any systemic immune response in animals exposed to the virus. Studies comparing the patterns of the systemic acute phase response in experiments with FMDV isolates of varying virulence have been initiated. Preliminary results indicate a close relationship between the timing and magnitude of the serum APP response with the severity of clinical disease, providing an objective tool for validation of severity of infection.

## Competing interests

The authors declare that they have no competing interests.

## Authors' contributions

CS planned and performed animal experiments and laboratory analysis of samples and drafted manuscript. PH contributed with protocols, reagents and instructions regarding assays for APPs, as well as advice on analysis and interpretation of results and critical revision of manuscript. AS performed statistical analysis of data and contributed to interpretation of results. KT helped with design and practical performance of animal experiments including assessment of clinical lesions in the animals. GB contributed with planning and coordination of the study, as well as advice on scientific content of the manuscript and critical revision of the text. All authors have read and approved the manuscript.
